# Seeing objects improves our hearing of the sounds they make

**DOI:** 10.1093/nc/niaa014

**Published:** 2020-08-09

**Authors:** Kingson Man, Gabriela Melo, Antonio Damasio, Jonas Kaplan

**Affiliations:** n1 Brain and Creativity Institute, University of Southern California, 3620A McClintock Avenue, Los Angeles, CA 90089, USA; n2 Institute of Basic Health Sciences, Federal University of Rio Grande do Sul, 500 Sarmento Leite Street, Porto Alegre, RS, 90050-170, Brazil

**Keywords:** multisensory integration, binding, imagery, psychophysics

## Abstract

It has been established that lip reading improves the perception of auditory speech. But does seeing objects themselves help us hear better the sounds they make? Here we report a series of psychophysical experiments in humans showing that the visual enhancement of auditory sensitivity is not confined to speech. We further show that the crossmodal enhancement was associated with the conscious visualization of the stimulus: we can better hear the sounds an object makes when we are conscious of seeing that object. Our work extends an intriguing crossmodal effect, previously circumscribed to speech, to a wider domain of real-world objects, and suggests that consciousness contributes to this effect.

## Introduction

Can seeing an object help us to hear better the sound that object makes? The answer might be yes, given that we can detect speech sounds more sensitively when we watch a speaker’s articulatory movements—when we lip read—especially in noisy environments (Grant and Seitz 2000; Bernstein *et al.* 2004). To date, however, visual enhancement of auditory sensitivity has only been demonstrated for linguistic objects, and there are reasons to believe that this crossmodal effect might be narrowly circumscribed. Audiovisual speech is regarded as a “special” class of stimulus and presumed to be processed in a privileged mode—i.e. there are behavioral advantages for recognizing speech stimuli as such (Tuomainen *et al.* 2005; Vatakis *et al.* 2008). It is not known if this remarkable crossmodal effect respects the hard boundary of linguistic objects or if it generalizes to a wider domain of natural objects.

There is a deep literature on the crossmodal interactions between simple tones and flashes of light. For these elementary audiovisual events, there is ample evidence of both crossmodal facilitation, in which a flash helps to detect a beep (e.g. Child and Wendt 1938; Schirillo 2011) and interference, in which a flash interferes with processing tones (e.g. Colavita 1974; Fassnidge *et al.* 2017). By comparison, material objects draw on semantic knowledge and permit much richer spectral and temporal crossmodal interchange than do beeps and flashes.

Here we report a psychophysical investigation into visual enhancement of auditory sensitivity, explicitly targeting objects that produce sounds other than speech. Under a variety of visual co-stimulation conditions, we estimated auditory detection thresholds with unbiased two-interval forced choice (2IFC) procedures. In a first study, we compared auditory thresholds obtained while subjects viewed a static fixation cross, with thresholds obtained while subjects viewed a video of the object. We also tested whether any enhancement provided by the object video was due to a reduction in temporal uncertainty (Tjan *et al.* 2014).

In a preregistered second study, we sought to replicate and extend the findings from the first. We performed a more stringent dissection of the temporal information provided by the object video, generating an abstract visualizer stimulus to provide fine temporal information with a minimum of object-related semantic information.

The binding together of sensory modalities is a critical feature of conscious experience. Accordingly, we also tested the dependence of crossmodal enhancement on conscious vision. Would the videos need to be consciously seen for auditory enhancement to occur? We manipulated conscious visibility with dichoptic presentation. Auditory thresholds were compared when subjects consciously saw the object video, and when the object video was rendered unconscious with continuous flash suppression (CFS, Tsuchiya and Koch 2005) or binocular rivalry (BR).

## Methods and Materials

### Study 1

#### Participants

We recruited 18 subjects from the community of the Universidade Federal do Rio Grande do Sul in Porto Alegre, Brazil. Subjects were adults with self-reported normal hearing and normal or corrected-to-normal vision. Subjects received no monetary compensation for their participation, following local regulations. Approval for research involving human subjects was obtained from the Brazilian Ministry of Health’s Ethics Commission and the Institutional Review Board of the University of Southern California.

#### Stimuli

Two sound-producing objects were used, a musical triangle and a tambourine. Audiovisual recordings of the objects generating sounds were made with a digital camera (Olympus E-P2). A set of sound stimuli and silent video stimuli was extracted from the recordings. All were truncated to 2 s.

The two other visual stimuli were a fixation cross and an onset cue. The onset cue was a fixation cross that shifted its color from white to red to white, in time with the onset and offset of the auditory signal.

All stimuli were presented in a light- and sound-attenuated testing chamber. Videos were displayed on a 14″ Dell monitor (60 Hz refresh rate), at a viewing distance of 40 cm. Sounds were played over Sennheiser HD-202 headphones driven by a Macbook laptop. Stimulus presentation was precisely controlled with Psychtoolbox (Version 3.0.10) (Brainard 1997) in Matlab (R2015b). Subjects individually adjusted volume levels to one that they would be comfortable with over long-duration testing. Subjects were familiarized with the audiovisual object stimuli by viewing them on a loop for 1 min.

**Figure 1.**
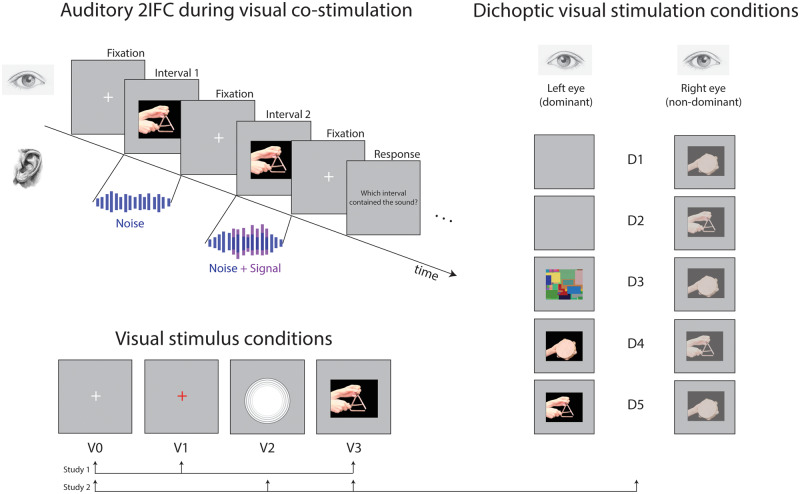
Schematic of experiments. Auditory thresholds were measured using a 2IFC task during co-presentation of visual stimuli. Left: Subjects viewed a computer monitor showing a white fixation cross at the start of each trial. During the two noise intervals, one of the following visual conditions occurred: (V0) no change in the fixation cross; (V1) the fixation cross changed color from white to red, (V2) a circle appeared and dynamically expanded and contracted in time with the sound amplitude; or (V3) the congruent object video was shown. Illustrated for the case of the triangle sound. Right: Under dichoptic viewing conditions, subjects wore goggles showing different images to each eye. For monocular conditions, the dominant eye was shown a blank screen while the nondominant eye was shown an object that was either congruent (D1) or incongruent (D2) with the sound. For the continuous flash suppression condition (D3), the dominant eye was shown the CFS stimulus and the nondominant eye was shown the congruent object. For the binocular rivalry condition, we presented one object in high contrast to the dominant eye, and the other object in low contrast to the nondominant eye. In this way, we controlled the contents of visual consciousness to either be congruent (D4) or incongruent (D5) with the sound. Illustrated for the case of the tambourine sound, with the left eye dominant.

We used a 2IFC task (see Fig. 1). Each trial contained two intervals of sound, one containing Gaussian white noise, the other containing the same noise sample with an additive acoustic signal. Noise duration was 4 s and signal duration was 2 s; signal onset was randomly placed between 1 and 2 s after noise onset. The stereo signal was averaged across L/R channels. To reduce temporal cueing by sudden sound onset/offset (auditory attack and release), the auditory signal was linearly faded in and out over 500 ms each.

#### Procedure

For each 2IFC trial the subjects indicated which interval contained the auditory signal. We adaptively estimated the threshold signal-to-noise ratio (SNR) for detection of an auditory signal embedded in noise. Noise level, set by each subject, was held constant; signal RMS power was varied to target 70.7% accuracy using the transformed up-down procedure (Levitt 1971) with a two-down one-up rule. The initial trial was presented at 0 dB SNR, with step sizes diminishing on the following schedule: 3 dB to Reversal (R) #2, 1 dB to R5, 0.5 dB to R8, 0.25 dB to R10, and 0.1 dB to R13. Threshold was calculated as the mean of the final 10 reversals.

The order of presentation of objects was counterbalanced across subjects. Thresholds were measured for the fixation cross condition, then the onset cue condition, and finally the object video condition.

#### Statistical analysis

We hypothesized that auditory thresholds would be lower when viewing some stimuli over others. Specifically, we tested the following directional hypotheses:

1. Object video < Fixation cross.
2. Object video < Onset cue.
3. Onset cue < Fixation cross.

We calculated the likelihood of obtaining differences across conditions greater than the ones observed, in an empirical distribution of values obtained under the null hypothesis (see Siegel and Castellan 1956). Under the null, visual conditions do not affect auditory threshold values and so may be freely permuted. Each subject’s thresholds were randomly reassigned among visual conditions 10,000 times, each time calculating the group average difference between visual conditions. For example, under Hypothesis 1, we randomly (with probability 0.5) swapped the thresholds measured when a subject viewed the object video and when a subject viewed the fixation cross. This was done for each subject and then the group average difference between the two conditions was calculated. This constituted one permutation of the experimental data. This procedure was repeated 10,000 times to build a null distribution of group average differences. Finally, the group average difference was calculated for the original, unswapped data. The *P*-value was calculated as the proportion of values in the null distribution equal to or greater than the value for the unpermuted data. This permutation procedure permits group-level inference without relying on assumptions of the shape of the distribution of thresholds, or of equal variance among subjects. We omit summary statistics of variation in favor of plots of individual-level data that illustrate the total observed variation.

*P*-values for all pairwise *t*-tests were corrected for multiple comparisons by holding the false discovery rate below 5% (Benjamini and Hochberg 1995). For nonsignificant results, we calculate the Bayes factor (Dienes 2014; using the calculator by Singh at https://medstats.github.io/bayesfactor.html). This determines the extent to which the data either support the null hypothesis or are instead not sensitive enough to distinguish between hypotheses. We apply the small sample size correction to SEM and use a uniform distribution between 0 and 5.

In a supplementary analysis, we used permutation methods to perform a repeated measures factorial ANOVA using the permuco package in R (Frossard and Renaud 2018; based on theory by Kherad-Pajouh and Renaud 2014). We modeled the effect of two factors, object type (containing the levels triangle and tambourine) and visual cue condition (containing the levels fixation cross, onset cue, and object video), on auditory threshold. All combinations of object type and visual cue were measured in each subject. This tested for an effect of object type on auditory thresholds, as well as for an interaction between object types and visual cue conditions on auditory thresholds.

### Study 2

In a preregistered second study, we used the Psi method of Bayesian adaptive estimation of psychometric thresholds (Kontsevich and Tyler 1999) and repeated testing of subjects (15 h over 5 days). The study plan, stimuli, scripts, and data may be freely accessed at the OSF project page: https://osf.io/8fyb2/. All comparisons in Study 2, with the exception of those labeled exploratory, were specified in the preregistration.

#### Participants

Subjects were recruited as above. Our sample size (*n* = 9) was informed by a prospective power analysis based on the effect size observed in Experiment 1 (Cohen’s *d* = 1.13). Setting alpha = 0.05 and power = 0.8, we required at least seven subjects. We recruited 10 subjects (including one of the authors, G.M.). One of the subjects dropped out, for an effective enrollment of nine.

#### Stimuli

The same object stimuli were used as above, though presented at a shorter duration of 1 s embedded within a 2-s noise interval, with signal onset randomly chosen between 0.5 and 1 s after noise onset. The signal was linearly faded in and out over 200 ms each.

A visualizer cue was created by using the amplitude envelope of the object sound to modulate the size of a circle, following Maddox *et al.* (2015). The auditory signal was passed through a zero-lag, fifth order, 60 Hz lowpass Butterworth filter, using the Matlab functions *butter* and *filtfilt*.

For dichoptic visual stimulus presentation, we used a head-mounted display consisting of a smartphone (Motorola G4, 5.5″ screen) placed in a TT-VR003 3D VR Headset (TaoTronics). The left and right halves of the screen were exclusively displayed to the left and right eyes. The smartphone screen was controlled as an external display with the TwomonUSB Android App (Easy&Light Software).

A CFS stimulus was created by rapidly translating and overlaying rectangles of random colors and sizes at ∼10 Hz (modified from http://martin-hebart.de/webpages/code/stimuli.html). A CFS stimulus of 120 s duration was pregenerated, from which 2 s segments were randomly extracted. We verified that the head-mounted display and software driver was capable of reproducing visual stimuli with correct timings by filming the display and counting frames, yielding a lower bound of 40 display updates per second.

Adapting the strategy of presenting dichoptically unbalanced stimuli in CFS, we exerted strong control of perceptual dominance during BR by presenting one object video to the subject’s dominant eye and at greater contrast, and the other object video to the nondominant eye at lower (5% luminance) contrast. The “dominant” object video was either congruent or incongruent with the sound. The following types of dichoptic visual stimuli were created:

- Monocular object: blank in the dominant eye, low-contrast object in the other.
- CFS-masked objects: CFS in the dominant eye, low-contrast object in the other.
- BR: high-contrast object in the dominant eye, low-contrast object in the other.

#### Procedure

Five repetitions of the thresholding procedure were performed, resulting in 15 h of psychophysical testing over 5 days for each subject. Subjects became familiarized with the audiovisual stimuli by viewing them on a loop for 1 min. The subject’s dominant eye was determined using a hole-in-card test (Handa *et al.* 2004). An initial auditory threshold was coarsely estimated with a one-down one-up adaptive procedure and constant step size of 1 dB for six reversals. This provided the Bayesian prior for the main thresholding procedure using the Psi method implemented in the Palamedes Toolbox (Prins and Kingdom 2009). Due to a software bug omitting the final (45th) trial from ∼10% of experimental sessions, we excluded it from all sessions and took the thresholds after 44 trials. Over the five repetitions of each thresholding session, a total of 220 trials contributed to each threshold estimate.

On each day of testing, auditory thresholds were estimated for both objects under the following free-viewing visual conditions: static fixation cross; congruent object video; amplitude envelope visualizer; and CFS. After a rest, auditory thresholds were taken with dichoptic visual presentation of the following conditions: monocular congruent object; monocular incongruent object; CFS of the congruent object; BR with congruent object dominant; and BR with incongruent object dominant. In CFS and BR conditions, subjects reported whether they could see the masked/nondominant stimulus after each trial. Subjects were instructed to set a liberal criterion and report seeing the lower contrast object if any of its features could be clearly seen and if visibility of the higher contrast object was at all impaired. If the masked stimulus was reported as seen, the trial was repeated. This occurred in 2% of CFS/BR trials.

On the final day, subjects rated the two object stimuli for their auditory and visual vividness. They also completed the Adapted Betts QMI Vividness of Imagery Scale (Sheehan 1967).

#### Statistical analysis

We performed permutation testing as above. Each day’s thresholds were entered into comparisons and permuted, but comparisons were never permuted across days. We also tested for a correlation between individuals’ vividness of sensory imagery and the magnitude of their crossmodal enhancement. Enhancement was calculated as the difference between auditory thresholds measured when viewing the fixation cross and when viewing the object video. We calculated the Pearson correlations between the auditory imagery subscale and enhancement, and between the visual imagery subscale and enhancement.

In a supplementary analysis that was not preregistered, we ran a repeated measures factorial ANOVA with permutation testing as for Study 1 above, but with the difference that Study 2 involved five repeated measures of all conditions within each subject. In addition to the preregistered pairwise comparisons of the visual cue conditions, we tested for an effect of object type on auditory thresholds, as well as for an interaction between object type with visual cue condition on auditory thresholds. We performed two separate ANOVAs, one for the free-viewing conditions and one for the dichoptic viewing conditions.

## Results

### Study 1

There was a significant main effect of visual cue on auditory threshold (*F*_2, 34_ = 17.1, *P *=* *0.0001). Below, we report the results of planned pairwise comparisons among the visual cues. There was a significant main effect of object type on auditory threshold (*F*_1, 17_ = 81.2; permutation *P* = 0.0001), with the triangle sound detected at lower thresholds than the tambourine sound. There was no significant interaction between object type and visual cue (*F*_2, 34_ = 0.54, *P *=* *0.59).

#### Object video vs. fixation

**Figure 2.**
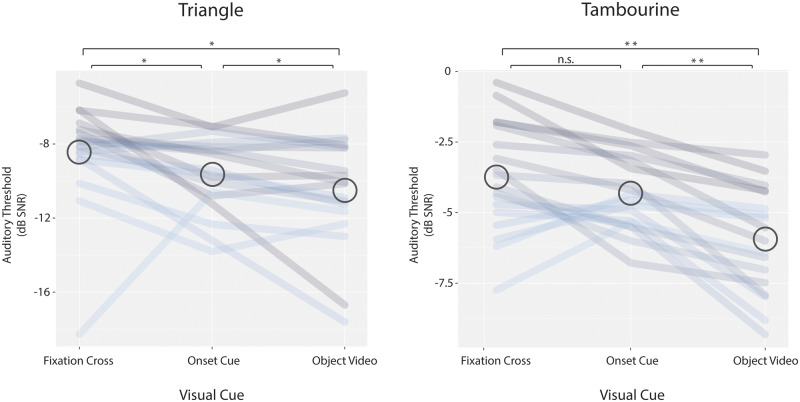
Visual cues reduced the detection thresholds for object sounds presented in noise. Each line joins the thresholds measured under three visual conditions for a particular subject. Lines are colored from gray to blue, sorted in descending order of threshold values in the fixation condition. Circles are centered on the group average threshold, but note that statistical comparisons were performed in a subject-wise manner. Auditory sensitivity was greater when viewing the object video than when viewing a static fixation cross. Object videos also yielded lower auditory thresholds than did a visual onset cue. Asterisks throughout denote the following permutation *P*-values: *<0.05; **<0.01; ***<0.001.

We found subject-wise improvement in auditory sensitivity (reduced detection thresholds) with co-presentation of object videos, as compared to co-presentation of a static fixation cross (Fig. 2; mean reductions of 2.04 and 2.21 dB SNR for the triangle and the tambourine, respectively; false discovery rate (FDR)-adjusted permutation *P*-values = 0.026 and 0.003).

#### Onset cue vs. fixation

We found a mixed pattern of auditory improvement by the co-presentation of a timing cue, as compared to presentation of a fixation cross alone. The timing cue significantly improved detection of the triangle sound (mean reduction of 1.2 dB SNR; *P *=* *0.048), but did not significantly improve detection of the tambourine sound (mean reduction of 0.58 dB SNR; *P *=* *0.051; Bayes factor *B *=* *0.6).

#### Object video vs. onset cue

For both objects, co-presentation of the object videos improved auditory sensitivity, as compared to the timing cues (mean improvement of 0.86 and 1.63 dB SNR for triangle and tambourine, respectively; *P *=* *0.038 and 0.003).

### Study 2: Free-viewing

In the free-viewing conditions, there was a significant main effect of visual cue on auditory threshold (*F*_2, 88_ = 13.3, *P *=* *0.0001). Below, we report the results of planned pairwise comparisons among the visual cues. There was a significant main effect of object type on auditory threshold (*F*_1, 44_ = 658.9; *P* = 0.0001), with the triangle sound detected at lower thresholds than the tambourine sound. There was no significant interaction between object type and visual cue (*F*_2, 88_ = 2.01, *P *=* *0.14). See Supplementary Data for the results of all planned comparisons stated in our pre-registration.

#### Object video vs. fixation

**Figure 3.**
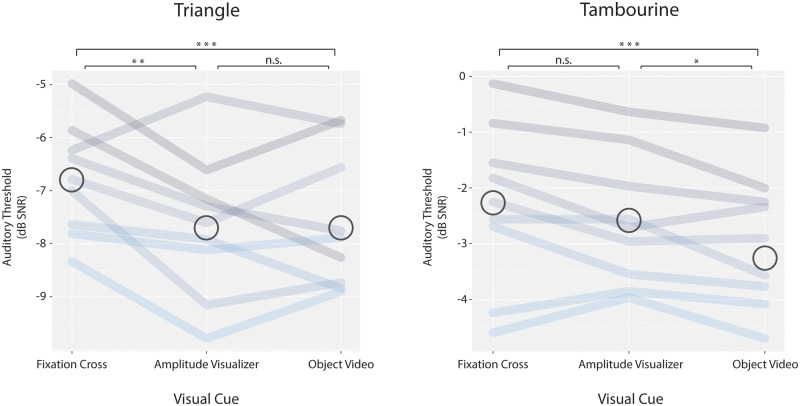
Replication of object-based visual enhancement of auditory detection. A preregistered follow-up study replicated the reduction in auditory detection thresholds by co-presentation of the corresponding object video, as compared to co-presentation of a fixation cross. For the triangle, a visualizer containing fine temporal information yielded lower auditory thresholds than did the fixation cross. For the tambourine, the object video was a superior cue to the visualizer.

We replicated the main finding from our first study: for both objects, co-presentation of an object video resulted in higher auditory sensitivity (lower thresholds) than co-presentation of a static fixation cross (Fig. 3; mean reductions of 0.81 and 0.65 dB SNR for triangle and tambourine, respectively; FDR-adjusted permutation *P*s = 0.0005 for both objects).

#### Visualizer vs. fixation

Once again, we found a mixed pattern of auditory enhancement by a visual temporal cue. Auditory sensitivity was significantly higher for detection of the triangle sound when accompanied by the visualizer (mean reduction of 0.87 dB SNR; *P *=* *0.0017), but not for the tambourine sound (mean reduction of 0.29 dB SNR; *P *=* *0.09; *B *=* *0.219).

#### Object *video vs. visualizer*

For the triangle, the object video did not significantly enhance auditory sensitivity in comparison to the visualizer (increase in thresholds of 0.05 db SNR; *P *=* *0.59; *B *=* *0.09). However, for the tambourine, the object video yielded significantly lower auditory thresholds than did the visualizer (0.36 dB SNR mean reduction; *P *=* *0.017).

#### Correlation between vividness of imagery and sensitivity enhancement

There were no significant subject-wise correlations between vividness of sensory imagery and magnitudes of crossmodal enhancement. The correlation between crossmodal enhancement and vividness of auditory imagery was *r* = 0.62 (*t* = 2.09, df = 7, *P* = 0.08). The correlation between crossmodal enhancement and vividness of visual imagery was *r* = 0.15 (*t* = 0.4, df = 7, *P* = 0.7).

### Study 2: Dichoptic Viewing

Under dichoptic viewing, there was a significant main effect of object type on auditory threshold (*F*_1, 44_ = 665; *P *=* *0.0001), with the triangle sound detected at lower thresholds than the tambourine sound. There was a significant main effect of visual cue, across the four types of dichoptically presented stimuli, on auditory threshold (*F*_3, 132_ = 6.1, *P *=* *0.0005). Below, we report the results of planned pairwise comparisons among the visual cues. There was no significant interaction between object type and visual cue (*F*_3, 132_ = 1.1, *P *=* *0.3460).

#### Masking the object video from consciousness with CFS

**Figure 4.**
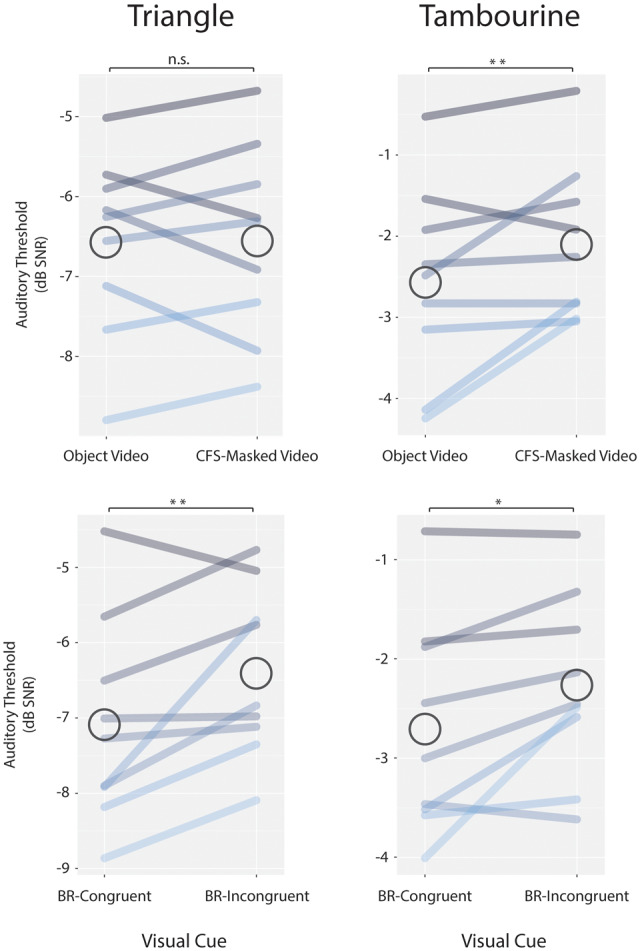
Visual enhancement of auditory sensitivity was modulated by visual consciousness. *Above*, for the tambourine sound only, masking the visually presented object from consciousness raised the auditory threshold, as compared to consciously viewing the object without CFS. *Below*, both objects were visually presented, one to each eye, to induce BR. Auditory thresholds were lower when BR cues resulted in visual experience of the congruent object, rather than of the incongruent object. This latter finding was an exploratory analysis not anticipated in our preregistration.

Auditory detection thresholds for the triangle were not significantly modulated by CFS (Fig. 4, top left; mean increase of 0.02 dB SNR; *P *=* *0.5; *B *=* *0.18). However, auditory thresholds for the tambourine were significantly higher when its video was masked by CFS (Fig. 4, top right; 0.47 dB SNR mean reduction; *P *=* *0.006).

#### Consciously seeing the congruent, vs. the incongruent, object under BR

This comparison was a follow-on, exploratory analysis not anticipated in our preregistration. The dominant object was reported to be in exclusive visual awareness in 98% of BR trials. For both sounds, auditory thresholds were lower when the congruent object was consciously visible than when the incongruent object was consciously visible (Fig. 4, bottom; mean reductions of 0.69 and 0.44 dB SNR for triangle and tambourine, respectively; *P *=* *0.003 and 0.024).

## Discussion

Our results demonstrate the equivalent of lip reading for nonlinguistic objects. Seeing an object helps us better hear the sound that the object makes. The observed magnitudes of visual enhancement for hearing object sounds (0.65–2.21 dB SNR) were comparable to those previously reported for improvement in hearing speech sounds (0.8–2.2 dB SNR; Grant and Seitz 2000). This improvement was not fully accounted for by the coarse or fine temporal information provided by abstract visual cues. Visual cues were more effective when they were consciously experienced than when they were presented to the eyes but not “seen.” When both object videos were presented, one to each eye, subjects were more sensitive to hearing an object’s sound if they consciously saw the congruent object. When an object video was abolished from consciousness using CFS, only the tambourine’s thresholds were affected. One possible explanation for this discrepancy between the manipulations of visual consciousness is the ∼10-fold greater depth of ocular suppression achieved by CFS compared to BR (Tsuchiya *et al.* 2006).

### Hearing better, not quicker

Our findings of auditory threshold reduction demonstrate the enhancement of hearing itself, and not the enhanced performance of a behavior informed by hearing. An example of behavioral enhancement is a reduction in reaction times with multimodal stimulation. For object recognition, it is known that co-presentation of an object’s sound and image can reduce reaction times compared to presentation in one modality alone, or presentation of incongruent objects across the modalities (Giard and Peronnet 1999; Molholm *et al.* 2004; Suied *et al.* 2009). But speeded reaction times do not necessarily reflect increased sensitivity. One may be quicker to report recognition of an object due to the summation of multimodal information, rather than the enhancement of one modality by the other.

Crossmodal improvements in reaction time and accuracy have also been observed when identifying objects specifically by their sounds (Schneider *et al*., 2008; Yuval-Greenberg and Deouell, 2009; Masakura *et al*., 2016). The effect may be long-lasting, as prior study of visual objects primes later identification of their sounds (Greene *et al.* 2001). However, these patterns of results can be explained by a visually mediated shift of an auditory criterion. A person might be quicker to report hearing a dog’s bark when primed with a picture of a dog, not necessarily because the bark sounds any clearer, but because they are more inclined to ascribe barking to the same noisy sound.

We show, in the 2IFC object detection setting, a basic, sensory-level improvement of hearing by seeing. Our experimental dissection of temporal information from the visual cue reveals the effect is at least partially mediated by semantic knowledge. We speculate that the mixed results from the two objects are due to an object- or event-dependence of the effect. Different objects have different levels of emphasis on their semantic content. Some objects may rely more on timing cues (e.g. our triangle) and others on semantics (e.g. our tambourine) for their crossmodal enhancement. Language objects are especially well-differentiated by their crossmodal semantics (though they are perhaps “not that special” after all, see Vroomen and Stekelenburg 2011). At the other end, the minimal audiovisual events of beeps and flashes may rely more on the structural aspects of temporal and spatial coincidence.

With respect to distinguishing sounds by their structural properties, the stationarity, or consistency, of a sound may play a role in its detection in noise. A recent study generated the illusion of hearing sound textures during several seconds of pure white noise (McWalter and McDermott 2019). The strength of the illusion was modulated by the stationarity of the sound; pure noise was more frequently mistaken, e.g., for applause (stationary) than for firecrackers (nonstationary). Future studies could test whether nonstationary sounds, being less amenable to statistical completion, would benefit more from the addition of a crossmodal cue.

### Imagery or attention?

An object-based visual cue provides rich temporal and semantic information that can automatically draw auditory attention (Molholm *et al.* 2007), and/or evoke a precisely specified auditory image. The question arises if one or the other mechanism is responsible for crossmodal enhancement. In the intramodal case, auditory imagery has been shown to enhance auditory sensitivity in a stimulus-selective manner (Farah and Smith 1983). A later study showed that auditory imagery can also selectively interfere with auditory detection (Okada and Matsuoka 1992). This argues that imagery, rather than attention, may be their operative mechanism. Imagery can become a competitor to the stimulus, whereas the recruitment of attention would not be expected to impair detection.

Earlier studies of auditory imagery have shown a Perky effect, where internal imagery is mistaken for an external stimulus (Perky 1910). If subjects can hear an imagined sound in their mind’s ear vividly enough to mistake it for a presented sound—as indeed our subjects often did, reporting hearing signals in both noise intervals even though they were strictly presented in only one (see McWalter and McDermott 2019)—then imagery may change the nature of the behavioral task. The addition of subjectively experienced imagery may transform an *objective signal detection* task into a *subjective intensity discrimination* task. The two intervals to be discriminated would be one containing [auditory imagery + noise] and the other containing [external signal + auditory imagery + noise]. Crossmodal facilitation may therefore occur because a visual stimulus triggers auditory imagery.

We showed that crossmodal sensitivity enhancement is modulated by subjective visual experience. Whichever object was consciously seen in the mind’s eye would have been the same one consciously heard in the mind’s ear. Indeed, there is evidence that simply imagining the sight of an object can affect auditory perception of the object (Berger and Ehrsson 2013). These examples of crossmodal imagery may be considered cases of crossmodal perceptual completion (Spence and Deroy 2013).

### Is consciousness necessary for crossmodal sensitivity enhancement?

We found enhancement of auditory sensitivity when a congruent object, rather than an incongruent object, was in visual consciousness. However, this does not imply that visual consciousness is required for visual-to-auditory enhancement. Unconscious vision—of a picture masked by CFS—is still capable of driving object-based visual attention (Chou and Yeh 2012). It is possible that unconscious vision could drive auditory attention. But we are aware of no unconscious priming method, visual or otherwise, that can elicit specific auditory imagery. If the auditory sensitivity enhancement observed in our study is mediated not by auditory attention but by auditory imagery, we would predict that the visual cue must reach the level of visual awareness to be effective.

In the domain of language, reaction times to auditory stimuli can be enhanced with invisible visual cues. Identification of an auditory spoken word was speeded by CFS-masked visual speech of the congruent word (Plass *et al.* 2014). There is even evidence for congruency priming of audiovisual letters and numbers when subjects are unaware of both sight and sound (Faivre *et al.* 2014). However, we have come across no evidence that invisible visual cues may enhance auditory sensitivity, rather than auditory reaction times. On balance, we believe that the crossmodal enhancement observed in our study is probably mediated by conscious imagery.

## Conclusions

In summary, we show that seeing an object improves hearing for the sounds made by that object. This generalizes an interesting effect, previously circumscribed to lip reading of speech, to a much wider domain of real-world objects. This crossmodal enhancement was modulated by conscious visibility of the stimulus: you can better hear an object when you can consciously see that object. We identify mental imagery as the likely mediator of these effects. Perhaps more significantly, we connect the phenomenon of consciousness—thought to be related to the global broadcast of semantic information (Baars 1997)—with functional consequences on the ability of one sensory modality to enhance the sensitivity of another.

## Author Contributions

K.M. conceived and designed the study with input from G.M. and J.K. G.M. collected behavioral data. K.M. and G.M. performed data analysis. K.M. drafted the manuscript with supervision from A.D. All authors discussed the findings and provided input on the manuscript.

## Supplementary Material

niaa014_Supplementary_Data
