# Supplementary material for: Seeing objects improves our hearing of the sounds they make
Source: Neurosci Conscious. 2020 Aug 9;2020(1):niaa014. doi: 10.1093/nc/niaa014 (PMC7415264; doi:10.1093/nc/niaa014)
Supplement: niaa014_Supplementary_Data [file niaa014_supplementary_data.docx]

Supplementary Results

We first report the results of all planned comparisons stated in our pre-registration. They are presented as specific hypotheses and results grouped under general questions.

*Question 1. Does seeing an object improve auditory perception for that object?*

Hypothesis 1. Watching an object producing a sound, as compared to watching a static fixation cross, will result in a lower auditory detection threshold for that sound.

Result 1. Comparing the object video condition to the static fixation cross condition, we observe reductions in thresholds of 0.811 dB (*P* < 0.0001) for the triangle sound and 0.649 (*P* < 0.0001) for the tambourine sound.

H2. Watching an incongruent object (producing a sound different from the one heard), as compared to watching a congruent object, will result in a higher auditory detection threshold.

R2. This hypothesis was tested on thresholds from the dichoptic stimulation phase. Monocularly presented objects in low contrast did not show a significant congruency effect on auditory thresholds (reductions of 0.2 dB and 0.29 dB SNR for triangle and tambourine; *P*s = 0.19 and 0.09).

*Q2. Does the visual cue need to depict the object, or will some other abstract visual-temporal signal suffice?*

H3. Watching an abstract temporal cue (a circle varying in size according to the amplitude envelope of the auditory stimulus), as compared to watching a static fixation cross, will result in a lower auditory detection threshold.

R3. For the triangle, the abstract cue resulted in a significantly lower threshold than did the fixation cross (0.869 dB; *P* = 0.0005). The tambourine’s observed reduction, 0.294 dB, was not significant (*P* = 0.07).

H4. Nevertheless, the abstract cue will not be as effective as the object cue at enhancing auditory perception; auditory thresholds for the former will be higher than for the latter.

R4. This hypothesis was not supported for the triangle, which showed a slight increase in threshold (0.06 dB; *P* = 0.589). However, the tambourine showed a significant reduction in threshold when comparing the object to the visualizer (0.355 dB; *P* = 0.01).

*Q3. Is visual consciousness required for visual enhancement of auditory perception?*

H5. Visual presentation of a congruent object in one eye, rendered unconscious by continuous flash suppression (CFS) presented to the other eye, will abolish the enhancement effect of H1. Viewing an object under CFS, as compared to viewing the object alone, will result in higher auditory thresholds.

R5. This hypothesis was supported for the tambourine sound (0.474 dB reduction; *P* = 0.003) but was not significant for the triangle sound (0.025 dB reduction; *P* = 0.453).

H6. Visual presentation of a congruent object in one eye, rendered unconscious by binocular rivalry (BR) via presentation of a higher contrast incongruent object to the other eye, will abolish the enhancement effect of H1. Despite presentation of the congruent object to one eye, BR with the incongruent object dominating visual consciousness will result in higher auditory thresholds compared to viewing the congruent object alone.

R6. This hypothesis was not supported, as the differences for both sounds were not significant (triangle, 0.174 dB, *P* = 0.247; tambourine, 0.303 dB, *P* = 0.053).

H7. Visual presentation of an incongruent stimulus in one eye, rendered unconscious by the presentation of a higher contrast congruent object in the other eye, will preserve the enhancement effect of H1. Despite presentation of the incongruent object to one eye, BR with the congruent object dominating visual consciousness will result in an auditory threshold no different from viewing the congruent object alone.

R7. This hypothesis was supported for both objects, with no significant differences detected between the conditions (triangle *P* = 0.981, tambourine *P* = 0.755).

*Q4. Do individual differences in vividness of imagery correlate with the strength of crossmodal enhancement?*

H8. There will be a positive correlation between vividness of auditory imagery and the crossmodal enhancement of auditory thresholds, across subjects. There will be no correlation between vividness of visual imagery and crossmodal enhancement.

R8. The first hypothesis was not supported, with no significant subject-wise correlation between auditory imagery scores and magnitude of enhancement, calculated as the difference between object video thresholds and fixation cross thresholds. The second hypothesis was supported, insofar as no significant relationship was found.

In a follow-on analysis, we addressed the question posed by Spence & Deroy (2014)^[[1]](#footnote-1)^ on crossmodal imagery: would visually-triggered auditory imagery be correlated to one’s intrinsic auditory imagery? We correlated subjects’ scores on the auditory subscale of the imagery questionnaire with their reports of the vividness of the auditory imagery triggered by the visual stimuli. There was no significant relationship.

1. Spence, C., & Deroy, O. (2013). Crossmodal Mental Imagery. In Multisensory Imagery (pp. 157–183). New York, NY: Springer New York. http://doi.org/10.1007/978-1-4614-5879-1_9 [↑](#footnote-ref-1)
